# Exploring the influencing factors of patients sending red packets and the necessity of doctor-patient red packet agreements

**DOI:** 10.3389/fpubh.2024.1476724

**Published:** 2025-01-14

**Authors:** Pingli Li, Lixiang Wu, Jie Zhang, Surong Xiang, Li Fang

**Affiliations:** ^1^Department of Gynecology, Clinical Medical College and Affiliated Hospital of Chengdu University, Chengdu, China; ^2^School of Public Health, West China Fourth Hospital of Sichuan University, Chengdu, China; ^3^Department of Otorhinolaryngology Head and Neck Surgery, Western Theater Air Force Hospital of PLA, Chengdu, China

**Keywords:** inpatients, red packets, influencing factors, informal payments, red envelopes

## Abstract

**Objective:**

To investigate the influencing factors of inpatients giving red packets to doctors and explore the necessity of doctor-patient red packet agreements.

**Methods:**

A questionnaire survey was conducted among inpatients and their families who were hospitalized in several hospitals in Chengdu from January to June 2023. The survey asked about the patients’ (or their families’) attitudes and opinions on whether it was necessary to give red packets to doctors during hospitalization.

**Results:**

The vast majority of patients (80.7%) thought that it was not necessary to give red packets to doctors, and 87.0% of patients had never given red packets. 59.7% of patients chose senior doctors as the recipients of red packets, and 90.0% of patients thought that it was necessary to give red packets to doctors in 3A-grade hospitals. Patients’ attitudes toward giving red packets were positively influenced by their education level and previous experience of giving red packets.

**Conclusion:**

Despite education level and previous experience of giving red packets were all positive influencing factors for patients giving red packets, the proportion of patients who had given or intended to give red packets was relatively low. The majority of patients believed that giving red packets was unnecessary, and they held the view that doctors would not treat them less actively if they did not receive red packets. The question of whether signing a red packet agreement is necessary is worth contemplating at this time.

## 1 Introduction

The red packets (*Hongbao* 红包) are informal payments that patients or their families give to doctors in exchange for better or quicker health services (1, 2). While traditionally associated with gift-giving customs during holidays or celebrations, in the healthcare context, red packets are sometimes given as a means to express gratitude or, in some cases, to secure more attentive medical care. They are widespread and harm the doctor-patient relationship in China. The “Agreement between Doctors and Patients Not to Accept or Send “Red Packets”“(referred to as the Doctor-Patient Red Packet Agreement) is a document issued by the National Health and Family Planning Commission of the People’s Republic of China to regulate the behavior and interaction between doctors and patients, create an honest medical practice environment, and build a harmonious doctor-patient relationship (3). However, the Doctor-Patient Red Packet Agreement has been controversial since its promulgation in 2014 (4). Some studies believe that signing a doctor-patient red packet agreement can enhance mutual trust between doctors and patients and harmonize the doctor-patient relationship (5). While other studies have found that this move is an insult to the doctor’s profession and a manifestation of disrespect for medicine; just relying on signing an agreement is purely a piece of paper to entertain oneself, and cannot completely cure the hospital’s red packet behavior (6). At the same time, it will have a negative impact on the development of medical technology and the improvement of doctors’ standards have had a negative impact (7). With the development and progress of society and the improvement of people’s health awareness, patients not only have higher requirements for rights such as curing the disease itself and achieving informed consent, but also hope to receive more care and respect from medical staff (8). This is one of the root causes of the current intensification of conflicts between doctors and patients. As the main body of China’s medical service system, medical institutions, especially public hospitals, play a role not only in providing medical services and protecting people’s health, but also in conveying medical culture and demonstrating industry ethics. However, some medical workers ignore professional ethics and engage in bad behavior and misconduct, causing the doctor-patient relationship to become increasingly tense. Therefore, research and discussion on the doctor-patient relationship will help promote the development and improvement of the medical and health industry and better protect people’s health. This study investigates patients’ attitudes about whether it is necessary to send red packets, explores the necessity of the continued implementation of the “doctor-patient refusal to accept red packets agreement,” analyzes the influencing factors of patients’ attitudes and their impact on the doctor-patient relationship, and proposes corresponding recommendations. The suggestions provide a theoretical basis for building a harmonious doctor-patient relationship and enhancing mutual trust between doctors and patients.

## 2 Methods

### 2.1 Source

Samples were selected from several public hospitals in Chengdu using a combination of simple random sampling and convenience sampling methods. Specifically, the random number table method was employed for initial sampling, while convenience sampling was used to choose participants from each hospital between January and June 2023. A questionnaire survey was then conducted with inpatients and their family members to gather their attitudes and opinions regarding the necessity of giving red packets to doctors during hospitalization. A total of 640 questionnaires were distributed, and 638 valid questionnaires were recovered, with an effective recovery rate of 99.69%.

### 2.2 Quality control

The first draft of the questionnaire was designed through interviews, brainstorming and expert consultation, and a pre-survey was conducted before the formal survey, and the questionnaire was adjusted based on the results of the pre-survey. All investigators must undergo unified training and pass the survey before they can participate in the investigation. During the investigation, investigators are required to strictly abide by the investigation plan and are not allowed to change the investigation location and objects at will. Each time a questionnaire is completed, the investigator will first check the completion status. Unqualified questionnaires will be supplemented or modified after on-site questioning or re-explanation. The data is double-entry, and computer logic error checking and data cleaning are performed on the entry results to obtain the final data.

### 2.3 Variable selection

This study takes whether patients think it is necessary to give red packets as the dependent variable, which is, respectively, not necessary and necessary, in order to analyze patients’ attitudes toward giving red packets during hospitalization and its influencing factors.

Based on the purpose of the research and the availability of indicators, this study mainly selected four categories of indicators to analyze patient attitudes: ① Sociodemographic indicators: It mainly includes three variables: gender, age, and educational background of residents. ② Past experience: Previous studies have shown that whether patients or family members have given red packets during previous hospitalizations and related experiences will affect the patient’s attitude toward giving red packets in the future (9). Therefore, this study includes “whether red packets have been given before” as an explanatory variable. ③ Recipient of a gift: It includes two variables: doctor level and hospital level. ④ Patients’ underlying perceptions of doctors: Since whether a patient chooses to give a red packet during hospitalization is more affected by his or her own subjective cognition, this article selects “whether the patient thinks the doctor will treat him attentively when the doctor does not receive the red packet” to reflect the impact of potential cognition on attitude. The variable selection and definitions are seen in Table 1.

**Table 1:** Variable selection and definition.

Variable category	Variable name	Characterizing variables	Definition
Explained variable	Manner	Is it necessary to send red packets during medical treatment?	Not necessary = 0, Necessary = 1
	Sociodemographic indicators	Gender	Male = 1, Female = 0
Explanatory variables		Age	Under 45 years old = 1, 45–60 years old = 2, Over 60 years old = 3
		Educational qualifications	Elementary and Junior high school =1, High school/vocational high school/junior college = 2, Undergraduate = 3, Graduate student and above = 4
	Past experience	Have you ever sent red packets?	Yes = 1, No = 0
	Recipient of a gift	Doctor level	Senior doctor = 1, Resident physician = 0
		Hospital level	Extra large tertiary hospital = 1, Ordinary tertiary hospital = 2, County hospital = 3, Township hospital = 4
	Patients’ potential perceptions of doctors	If you do not receive the red packet, do not worry about treatment.	Won’t = 0, Probably = 1

### 2.4 Research methods and model construction

SPSS25.0 software was used to statistically describe demographic characteristics, and classified data were expressed as relative numbers. The dependent variables in this article are two situations where patients think it is unnecessary and necessary to send red packets, which is a discrete choice problem. The logit regression model is an effective model for multiple regression of binary categorical variables as the dependent variable. Therefore, this article uses the binomial logit model for modeling analysis. The patient’s attitude toward sending red packets is Y, “necessary” is defined as Y = 1, and “no” “Necessary” is defined as Y = 0, and the model is as follows, see Equation 1 (10):


(1) $$Y=\beta_{0}+\sum \beta_{i}X_{i}+\varepsilon$$

The probability that it is necessary to send red packets is recorded as P, and the probability that it is not necessary to send red packets is 1-P. β_0_ is the constant term, β_i_ is the regression coefficient of the independent variable, X_i_ is the independent variable, and *ε* is the random error term. After number conversion can get Equation 2 as follows (11):


(2) $$Ln\left(\frac{p}{1-p}\right)=\beta_{0}+\sum \beta_{i}X_{i}+\varepsilon$$

## 3 Results

### 3.1 Statistical description of patient (family) samples

Among the patient samples, 515 people (80.7%) thought it was not necessary to give red packets to doctors, and 123 people (19.3%) thought it was necessary to give red packets; including 349 men (54.7%) and 289 women (45.3%); In terms of age, young people under 45 years old account for the largest proportion (72.9%), followed by 45–60 years old (accounting for 24.0%); older adults over 60 years old account for 3.1%. In terms of academic qualifications, universities account for the largest proportion (69.0%), high schools account for 13.5%, graduate students and above account for 11.4%, and primary and junior high schools account for 6.1%, respectively. Judging from past experience, 87.0% of patients have never given red packets, 13.0% of patients have occasionally given red packets. Regarding the recipients of gifts, 59.7% of patients chose senior doctors, and 90.0% of patients believed that it is necessary to give red packets to doctors in 3A-grade hospitals (Extra large tertiary hospital + Ordinary tertiary hospital). In terms of patient perception, 69.3% of patients believed that doctors would provide attentive care even without receiving red packets, 30.7% thought that doctors might not provide attentive care without receiving red packets (see Table 2).

**Table 2:** Basic information about the sample.

Project	Classification	Count	Proportion (%)
Is it necessary	No need	515	80.7
	Is necessary	123	19.3
Gender	Male	349	54.7
	Female	289	45.3
Age	Under 45 years old	465	72.9
	45–60 years old	153	24
	60 and above	20	3.1
educational	Primary and Junior high school	39	6.1
	High school	86	13.5
	University	440	69
	Graduate students and above	73	11.4
Have you ever sent red packets?	no	555	87
	yes	83	13
Choose the level of doctor you want to send gift to	Resident physician	257	40.3
	Senior doctor	381	59.7
Select the hospital level for gift giving	Extra large tertiary hospital	452	70.8
	Ordinary tertiary hospital	122	19.2
	County hospital	46	7.2
	Township hospital	18	2.8
Do not pay attention to treatment if you have not give the red packet	Won’t	442	69.3
	Maybe	196	30.7

### 3.2 Model testing

In the logit regression model, the likelihood ratio test is generally used to test the significance of the independent variables and the dependent variables to determine whether the independent variables have the ability to explain the dependent variables and whether the model is practical. The likelihood ratio statistic approximately obeys the χ^2^ distribution. If the χ^2^ value of the model corresponds to the *p* value and is statistically significant, the null hypothesis is rejected, indicating that the information provided by the independent variables helps to better predict whether the event will occur (12). The χ^2^ value of the Logit model fitting in this article is 170.34, *p* < 0.05, which can be considered as valid model fitting (Table 3).

**Table 3:** Model testing results.

Model	−2 times the log-likelihood	χ^2^	df	*p*	AIC value	BIC value
Intercept only	625.55					
Final model	455.21	170.34	7	0	471.21	506.877

### 3.3 Regression results and analysis

**Figure 1:**
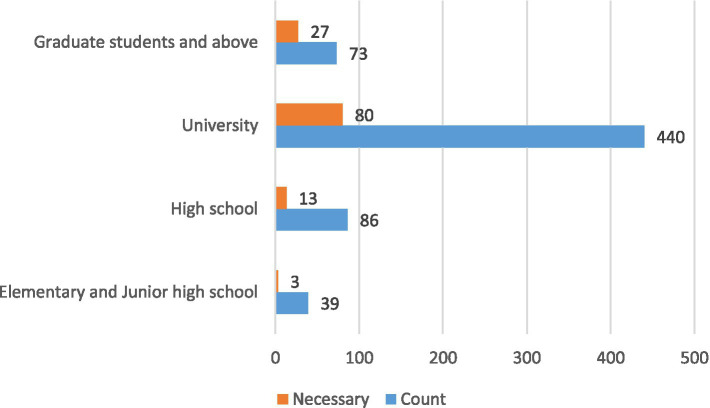
The histogram of the relationship between educational qualifications and the perception of the necessity of giving red packets.

The results revealed that the regression analysis of the patient’s educational background and past experience with giving red packets yielded statistically significant findings (*p* < 0.05). Using primary and junior high school as the dummy variable, the regression coefficients for university (1.777) and postgraduate and above (2.717) were positive, indicating that higher education levels positively influence attitudes toward the perceived necessity of giving red packets during hospitalization. Similarly, patients who had previously given red packets were more likely to do so in future visits. Using town hospital as the dummy variable, the regression coefficient for ordinary tertiary hospital (−1.351) was positive, indicating that scale of the hospital may positively influence attitudes toward the perceived necessity of giving red packets during hospitalization. In contrast, factors such as gender, age, patients’ perceptions of doctors, and the doctor level showed no statistically significant impact on patients’ attitudes toward giving red packets (Table 4). To better illustrate the relationship between education level and the perceived necessity of giving red packets, a histogram was created (Figure 1).

**Table 4:** Binomial logit regression results.

Variable type	Variable name		Regression coefficients	std.Err	*p*-value
	Intercept		−4.155	0.959	0.001*
Sociodemographic indicators	Gender	Male (female)	−0.045	0.249	0.921
	Educational qualifications	High school (Primary and Junior high school)	1.284	0.785	0.133
		University (Primary and Junior high school)	1.777	0.727	0.015*
		Graduate students and above (Primary and Junior high school)	2.717	0.784	0.001*
	Age	45–60 years old (Under 45 years old)	0.256	0.303	0.38
		60 and above (Under 45 years old)	−0.027	1.042	0.926
Past experience	Have you ever sent red packets?	Yes (no)	3.260	0.313	0.000*
Gift object	Doctor level	Senior doctor (Resident physician)	0.381	0.261	0.176
	Hospital level	Extra large tertiary (Township hospital)	−0.801	0.708	0.177
		Ordinary tertiary hospital (Township hospital)	−1.351	0.652	0.038*
		County hospital (Township hospital)	−0.476	0.593	0.502
Patients’ potential perceptions of doctors	Do not pay attention to treatment if you have not received the red packet from the patient	Maybe (will not)	−0.247	0.273	0.372

## 4 Discussion

The current situation of the doctor-patient relationship is a lack of trust and understanding between doctors and patients. In recent years, the doctor-patient relationship has become increasingly tense, and doctor-patient conflicts have become increasingly intensified. According to 2021 Chinese Physician Survey Report, it shows that more than half of medical staff do not want their children to apply for medical schools (13). The main reasons for the contradiction in the doctor-patient relationship are that hospitals focus on procedural services but not humanistic care, and medical staff focus on objective operations such as instrument inspection and treatment during diagnosis and treatment, which inadvertently “materializes” patients (14, 15). In addition, poor communication between doctors and patients, underutilization of doctors’ professional knowledge and skills, and the inability to protect patients’ rights and interests are also contributing factors to the tense doctor-patient relationship. Another important reason is that due to the relative shortage of medical resources and the often complex and changeable diseases, patients are in a relatively weak position during the medical treatment process (16, 17). In order to gain an active position in the doctor-patient relationship, patients often seek to send red packets to doctors (18). The “red packet phenomenon” is, at its root, a form of corruption. According to the Global Corruption Barometer (GCB) 2023 by Transparency International, China ranks 76th in the world with 42 points. Data from the GCB indicates that about 26% of Chinese respondents reported making informal payments in healthcare, which is higher than the global average of 11%. In contrast, countries like Germany and the United States report much lower rates, typically under 5%. Countries in Eastern Europe and Central Asia exhibit rates similar to China, where informal payments are more normalized due to systemic issues in healthcare delivery. This phenomenon, on the contrary, leads to the deterioration of the doctor-patient relationship. In order to curb the phenomenon of sending red packets, the National Health and Family Planning Commission promulgated the “Agreement on Not Accepting and Not Giving “Red Packets” to Doctors and Patients in 2014” (19). In November 2021, the National Health Commission, the National Medical Insurance Administration, and the State Administration of Traditional Chinese Medicine formulated and promulgated the “Integrity Practice of Medical Institutional Staff” based on the “Nine Precautions” in response to outstanding issues that have been strongly reported by the public in the current medical and health field (20). This guideline is a foundational document on integrity for all staff members of medical institutions. Article 8 clearly states: “Foster harmonious relationships, do not accept red packets from patients, adhere to medical ethics, and maintain strict self-discipline.” To understand the impact of the red packet agreement, we conducted a survey from the patients’ perspective.

Our survey results show that 80.7% of patients think it is not necessary to give red packets to doctors, and 87.0% of patients have never given red packets, 13.0% of patients have occasionally given red packets. Many patients and their families have never even heard of the “Doctor-Patient Red Packet Agreement,” and the atmosphere during the signing of the agreement is often weird. Many patients and family members inevitably have questions in their minds: Should we give red packets or not? Doctors in charge often need to spend more time and energy explaining the original intention of the “doctor-patient red packet agreement” to patients. Regarding the recipients of gifts, 59.7% of patients chose senior doctors, and 90.0% of patients believed that giving red packets to doctors is necessary in 3A-grade hospitals (China’s Hospital Grading System is a hierarchical classification used to organize healthcare resources and ensure quality control. Hospitals are divided into three main levels: Primary (Level 1), Secondary (Level 2), and Tertiary (Level 3), with each level further subdivided into Grade A, B, and C based on the hospital’s size, medical technology, and service capacity. 3A Hospitals (Top-Tier Tertiary Hospitals): These are the highest-grade hospitals in China, known for having the most advanced medical equipment, specialized departments, and highly skilled senior doctors. They often serve as regional medical centers for the treatment of complex and severe cases. In the 3A hospitals, they are divided into ordinary 3A hospitals and extra large 3A hospitals, the latter are stronger, generally the best hospitals in a province. In the classification of medical titles in China, senior doctors often refer to attending doctor or above, such as deputy chief physicians and chief physicians). Although the regression analysis results lack statistical significance, it is not difficult to see that the core of medical resources is concentrated in extra large tertiary hospitals and expert professors. The relative scarcity of these resources exposes the contradiction between the existing level of medical resources and the growing medical needs of the people (17). Chinese medical authorities have long implemented relevant policies to alleviate the uneven development of regional medical standards, such as policies related to doctors going to the countryside and further training before promotion (21, 22). The vast majority of patients (69.3%) trust doctors, and they believe that doctors will treat them attentively even without receiving red packets. Nearly 30% of the patients had doubts and believed that the doctor might not pay attention to treatment because they did not receive the red packet. Further through regression analysis, we found that educational qualifications and previous experience of giving red packets all have a positive impact on patients’ attitude toward giving red packets. Research by Kong XJ and others found that patients with higher education levels are more likely to give red packets. Compared with rural patients, urban patients are more likely to give red packets (9).

Although educational qualifications and previous experience of giving red packets are all positive factors that influence patients to give red packets, the proportion of patients who have ever given red packets and who have the intention to give red packets is relatively small. Whether it is necessary to sign a red packet agreement is still a question worth thinking about today. The red packet agreement is not binding on patients. In previous cases involving doctors receiving red packets, no punishment or penalty was imposed on the behavior of sending red packets. Patients and their families only had to face receiving red packets returned by doctors, waiting to be pacified by the health administrative department and the hospital, and for the best medical resources (1). Since the agreement is not legally binding for patients, its significance for both patients and doctors becomes questionable. Throughout the world, it is not uncommon to see red packets given at medical appointments, both in developed and developing countries (23–25). However, the red packet agreement exists only in China. We oppose doctors using their medical resources for personal gain and support strict measures against corruption involving doctors accepting bribes. But we have to admit that the phenomenon of giving and receiving red packets is only an individual behavior and is not widespread. In reality, whether the actual effect of the red packet agreement is to restrain doctors or to insult doctors has become a question that has to be answered. A harmonious doctor-patient relationship requires mutual respect, understanding and trust between doctors and patients. Doctors should respect the patient’s personal dignity, respect the patient’s right to know, autonomy and privacy, respect the patient’s opinions and suggestions, and meet the patient’s needs as much as possible. Patients should respect the doctor’s professional dignity, respect the doctor’s professional knowledge and skills, respect the doctor’s opinions and suggestions, actively cooperate with the doctor’s treatment and care, and constantly improve their self-care awareness and health literacy.

## Data Availability

The original contributions presented in the study are included in the article/supplementary material, further inquiries can be directed to the corresponding author.
